# Exploring exercise interventions in substance abuse treatment: A comprehensive bibliometric analysis

**DOI:** 10.1097/MD.0000000000041018

**Published:** 2024-12-20

**Authors:** Jiawei Chen, Liu Sun, Tatjana A. Shilko, Ying Tian, Jiawen Li, Qingyuan Wang, Xing Wang, Xiaolou Tian, Linan Zhang

**Affiliations:** a College of Sports Science, Shenyang Normal University, Shenyang City, Liaoning Province, China; b Faculty of Physical Education, National Research Tomsk State University, Tomsk, Russia; c Department of Rehabilitation Medicine, Anshan City Changda Hospital, Anshan City, Liaoning Province, China; d Shanghai Sport University, Shanghai, China; e Hunan Traditional Chinese Medical College, Zhuzhou City, Hunan Province, China.

**Keywords:** bibliometrics, exercise, substance abuse, visualization analysis

## Abstract

This study aims to comprehensively evaluate the research landscape related to exercise and substance abuse over the past 2 decades. A systemic bibliometric analysis was conducted using 2 powerful tools, the Bibliometrix package for R and VOSviewer software. The analysis covered a corpus of literature indexed in the Web of Science’s core collection. The publication counts related to exercise interventions within the context of substance abuse from 2004 to 2023, key journals extensively publishing research on this topic, institutional affiliations contributing significantly to this field, frequently co-occurring keywords to highlight research focal areas, and citation patterns for these studies were examined. The analysis identified a total of 2110 articles during the specified period. Notably, there has been a discernible upward trend in publication volume within this field over the past 2 decades. Prominent journals featuring extensive research on this topic include Drug and Alcohol Dependence, Frontiers in Psychiatry, and International Journal of Environmental Research and Public Health. Among the notable contributors to the field are researchers Greer TL and Trivedi MH. The University of California’s system has also emerged as a leading institution, fostering collaborations with diverse research entities. The most frequently recurring keywords were exercise, substance abuse, substance use disorders, mental health, and depression, among others, highlighting the research focus. This study offers insights and recommendations for future research in the area of exercise in substance abuse, emphasizing the need to explore physiological mechanisms and psychological comorbidities to optimize exercise as a therapeutic intervention.

## 1. Introduction

Substance abuse has emerged as an alarming global public health concern, jeopardizing both social well-being and harmony. According to the World Drug Report 2023,^[1]^ drug use escalated significantly over the past decade in 2021, more than 296 million people worldwide used drugs, reflecting a 23% increase from the previous decade. Simultaneously, the number of individuals with substance use disorder (SUD) surged to 39.5 million, which is a 45% increase over the same period. The widespread abuse of addictive substances poses substantial economic and health burdens, impacting not only individual health but also placing strain on families and societies. Social consequences, including divorce, crime, and unemployment, further exacerbate the issue.

Presently, 2 primary treatment modalities exist, drug withdrawal and nondrug withdrawal. Drug withdrawal, commonly employed in clinical practice, involves mild detoxification symptoms but heavily relies on therapeutic drugs. Traditional drug addiction treatment predominantly uses methadone, buprenorphine, and other detoxification instead of addictive drugs.^[2]^ However, these treatments inadequately address addicts’ psychological cravings and carry risks. For example, methadone can lead to addiction, and buprenorphine has a capping effect.^[3]^ Unfortunately, no specific treatment exists for synthetic drugs and symptomatic treatment strategies prevail. Nondrug interventions, including psychotherapy and exercise therapy, complement the treatment landscape.^[4,5]^

Previous studies highlight exercise therapy’s crucial role in aiding substance abuse withdrawal.^[6]^ Over the past 2 decades, exercise interventions have demonstrated protective effects across addiction stages, including initiation, maintenance, withdrawal, and relapse.^[7–10]^ Exercise-based interventions offer mild withdrawal symptom management, with advantages such as low cost, safety, and convenience. Consequently, exercise has become an effective strategy in substance abuse treatment.

An in-depth exploration of exercise’s impact on substance abuse can elucidate the mechanisms of withdrawal and recovery. By clarifying this relationship, researchers can identify new directions and treatment targets. While existing literature reviews summarize the research progress, they lack a comprehensive view of knowledge structure, dynamic trends, and critical research areas.^[11,12]^ Additionally, no bibliometric analysis has specifically examined exercise-related substance abuse research. The present study utilizes bibliometric methods to analyze and summarize academic research on exercise in substance abuse using the Web of Science database over the last 2 decades (2004–2023) to address this gap. This study’s objectives include identifying the current research status, hotspots and trends in the field, as well as findings research directions and research gaps in the field of exercise intervention as a complementary remedy for drug addiction therapy.

## 2. Research methodology

This study comprises 3 main components, performance analysis, citation analysis, and social structure and evolution. The performance analysis used annual scientific output, relevant sources, source growth, and author productivity to understand the current global status of “exercise in substance abuse” research. The research foundation in this field was established by tracking highly cited local references. These influential works provided essential context for this study. Collaborative networks among authors and national co-operations reveal distinct research camps, their respective research directions, and interconnections. Additionally, keyword co-occurrence analysis, trending topics, thematic map, and thematic evolution shed light on theme development over time. These insights predict future research trends and guide research investigations. The overarching goal is to advance substance abuse research by bridging knowledge gaps and leveraging bibliometric insights.

### 2.1. Search strategy

**Figure 1.**
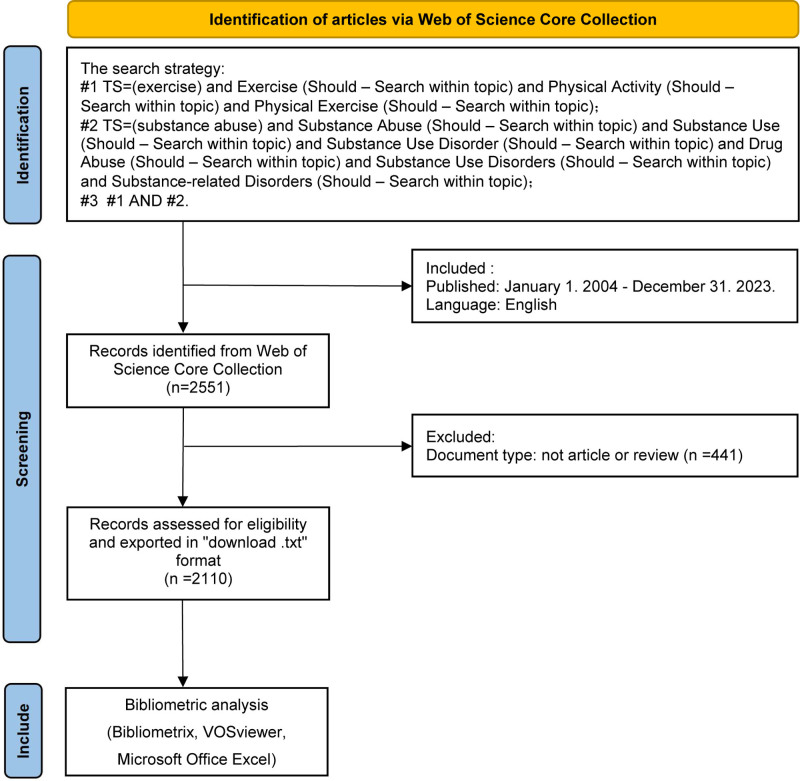
Research flowchart.

Data were retrieved from the Web of Science core collection, which is a comprehensive database, especially in the fields of natural sciences and medicine.^[13]^ This database encompasses 4 prominent citation indexes – Science Citation Index Expanded (SCIE), Social Sciences Citation Index (SSCI), Arts & Humanities Citation Index (A&HCI), and Emerging Sources Citation Index (ESCI) – and covers over 10,000 prestigious international scientific journals known for their significant impact factors.^[14]^ WoS has a set of strict evaluation standards for the inclusion of journals, and requires high editorial rigor and quality, thus enjoying a good reputation and trustworthiness in the academic community. The decision to focus on WoS for our search was driven by its capacity to facilitate comprehensive research endeavors. Use the search strategy:#1 TS = (exercise) and exercise (Should – Search within topic) and physical activity (Should – Search within topic) and physical exercise (Should – Search within topic); #2 TS = (substance abuse) and substance abuse (Should – Search within topic) and substance use (Should – Search within topic) and SUD (Should – Search within topic) and drug abuse (Should – Search within topic) and SUDs (Should – Search within topic) and substance-related disorders (Should – Search within topic); #3 #1 AND #2. Time span = January 1, 2004, to December 31, 2023. The search was conducted on January 04, 2024. The language is limited to English, and the document types are set to “Article” and “Review,” as shown in Figure 1. Download a complete record of each article, including title, abstract, keywords, year of publication, author, nationality, journal name, research direction, publisher, funding agency, references, and more.

### 2.2. Data analysis

The Bibliometrix (https://www.bibliometrix.org) software package was used to perform literature and visual analyses in *R*-4.2.2. The downloaded literature information from the Web of Science core collection was imported into the software and analyzed for the statistical stage, and scientific literature index, and complete a set of literature information analysis and visualization results display.^[15]^ This was done to identify relevant literature, to have a quick understanding of classic literature and leading figures, theme evolution, analysis of future trends.

Apart from Bibliometrix, this study also used VOSviewer, a visualization tool developed by Van Eck and Waltman in 2010 for the analysis of bibliometric networks.^[16]^ The VOSviewer was used to better visually present the association between exercise and substance abuse (version 1.6.18, https://www.vosviewer.com/) Additionally, the co-occurrence of keywords and authors’ cooperation network was visualized using the VOSviewer (Fig. 1).

## 3. Results

### 3.1. The annual number of papers published in the relevant literature

**Figure 2.**
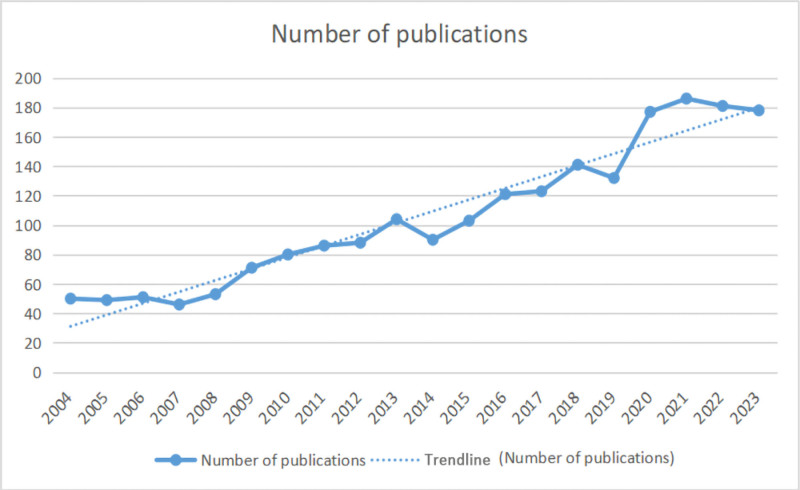
Distribution of publications by years.

A total of 2110 articles from 10,378 authors, 2795 institutions, 1060 journals, and 89 countries or regions were included in the study. The number of published papers exceeded 100 per year from 2015 to 2023. An overall growth was observed from 2004 to 2021, while a downward trend was found after 2021 (Fig. 2).

### 3.2. Most productive and influential authors

**Figure 3.**
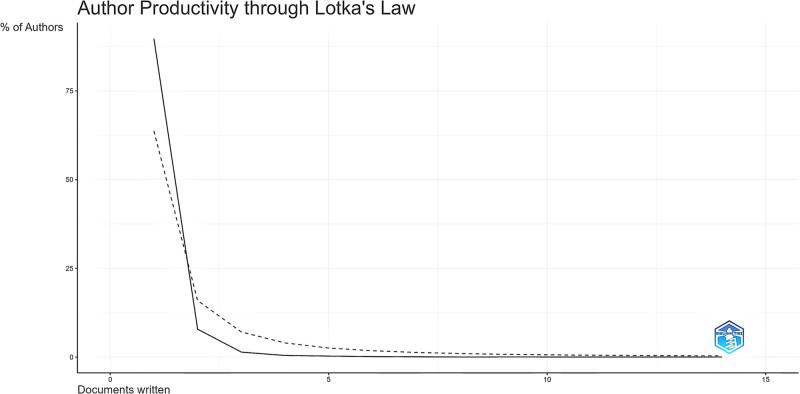
Distribution of authors with publications on exercise in substance abuse according to Lotka law.

The most productive and influential authors in the field of exercise research in substance abuse are listed according to the number of papers produced and the number of citations, as shown in Table 1. The most prolific authors of the exercise intervention study in substance abuse were Greer TL and Trivedi MH. Zhou CL is the most locally cited author. We also conducted a study on the productivity of authors according to Lotka law. Lotka law expresses the distribution of authors’ contributions to a particular literature in a particular period.^[17]^ Figure 3 shows the distribution of author productivity levels in the literature on exercise in substance abuse as determined by this law. The results of the analysis of author productivity according to Lotka law showed that 90.2% (n = 9364 authors) had published 1 article on physical activity in substance abuse, 7.1% (n = 737 authors) had published 2 articles, and 1.6% (n = 162 authors) had published 3 articles.

**Table 1:** Author rankings by number of publications and local citations (n = 10,378).

Author	Number of publications	%	Number of local citations
Greer TL	16	0.8	65
Trivedi MH	16	0.8	65
Rethorst CD	13	0.6	41
Walker R	13	0.6	55
Zhou CL	12	0.6	152
Hallgren M	10	0.5	47
Kelly PJ	9	0.4	34
Smith MA	9	0.4	110
Sussman S	9	0.4	31
Thanos PK	9	0.4	36

### 3.3. Most influential research

Citations in bibliometric analysis can be categorized as either local or global citations. Local citations refer to those from domain-specific studies included in the bibliometric analysis, s serve as a reference for the local citation category, while the global citation category includes all citations in the WoS (Web of Science). Both local citations and global citations are considered to provide a comprehensive assessment of the academic value and influence of the literature under study. Local citations emphasis the impact of the literature within the specific field of study to which it belongs, while global citations can help us to identify literature which may have had a significant impact in a number of different fields. Over the past 20 years, according to local citations, the ten most influential articles^[18–27]^ are listed in Table 2. The most cited local study is a meta-analysis on the impact of exercise on SUDs published by Wang et al.^[18]^ Additionally, an animal experimental study by Greenwood et al,^[27]^ which focuses on the reward mechanism of exercise on the mesolimbic brain, stands as the most globally cited literature.

**Table 2:** Articles with the highest number of citations.

No.	Author	Year	Article title	Local citations	Global citations
1	Wang D, et al^[18]^	2014	Impact of physical exercise on substance use disorders: a meta-analysis	68	160
2	Linke SE, et al^[19]^	2015	Exercise-based treatments for substance use disorders: evidence, theory, and practicality	54	100
3	Zschucke E, et al^[20]^	2012	Exercise and physical activity in the therapy of substance use disorders	51	103
4	Smith MA, et al^[21]^	2008	Aerobic exercise decreases the positive-reinforcing effects of cocaine	45	122
5	Buchowski MS, et al^[22]^	2011	Aerobic exercise training reduces cannabis craving and use in nontreatment-seeking cannabis-dependent adults	43	90
6	Dolezal BA, et al^[23]^	2013	Eight weeks of exercise training improves fitness measures in methamphetamine-dependent individuals in residential treatment	36	59
7	Roessler KK, et al^[24]^	2010	Exercise treatment for drug abuse – a Danish pilot study	33	58
8	Muller AE, et al^[25]^	2015	Group exercise to improve quality of life among substance use disorder patients	33	55
9	Wang D, et al^[26]^	2015	Acute exercise ameliorates craving and inhibitory deficits in methamphetamine: An ERP study	32	66
10	Greenwood BN, et al^[27]^	2011	Long-term voluntary wheel running is rewarding and produces plasticity in the mesolimbic reward pathway	30	257

### 3.4. Most relevant journals

The journals with the highest outputs are Drug and Alcohol Dependence, Frontiers in Psychiatry, and the International Journal of Environmental Research and Public health (Table 3). However, it is important to note that high research output does not equate to high academic influence on this topic.

**Table 3:** The 10 most relevant journals in the field of exercise research in substance abuse.

No.	Journal title	Number of publications	Impact factor (2023)	JCR (2023)
1	Drug and alcohol dependence	34	3.316	Q1
2	Frontiers in psychiatry	33	2.625	Q1
3	International journal of environmental research and public health	31	2.518	Q1
4	PLOS 1	29	2.429	Q1
5	Psychopharmacology	20	2.954	Q2
6	BMJ open	18	1.980	Q1
7	Frontiers in psychology	17	2.132	Q1
8	Salud mental	17	0.700	Q3
9	BMC public health	16	2.931	Q1
10	Journal of substance abuse treatment	16	3.114	Q1

### 3.5. Author collaboration network

**Figure 4.**
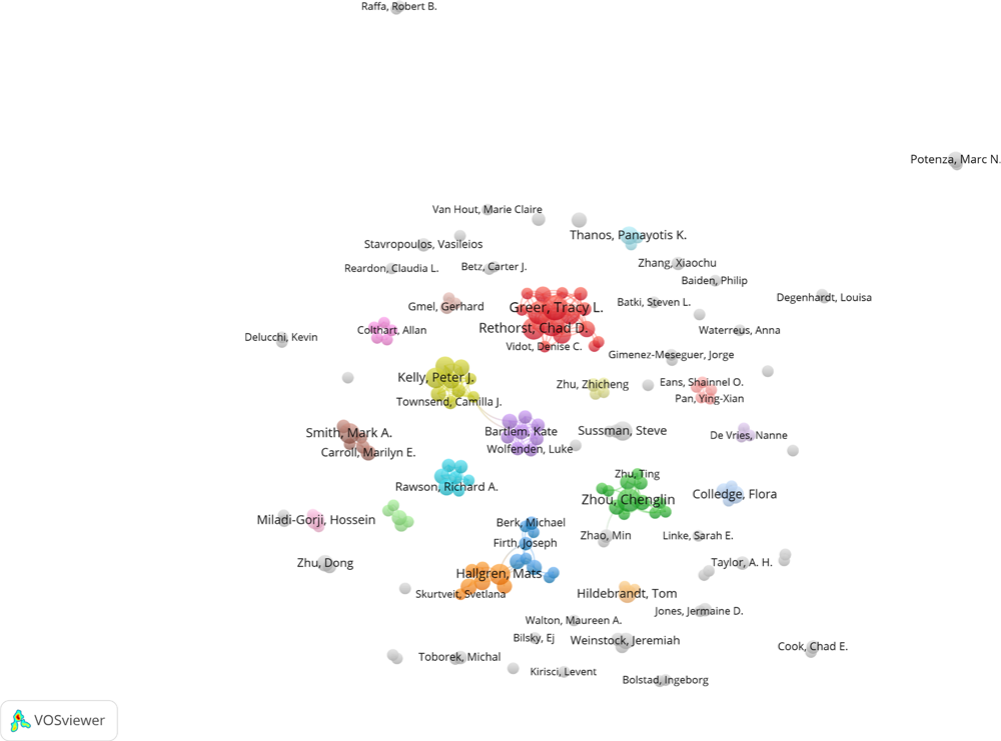
Co-authorship analysis (author level).

The author’s collaboration network in the field of exercise research in substance abuse is depicted in Figure 4. In this diagram, each circle represents an author and the size of the circle corresponds to the number of coauthored documents. The thickness of the lines between the circles represents the strength of the collaboration between the authors. All coauthors on the map belong to the same cluster. The results of the analysis show that Greer TL and Trivedi MH have significant correlations with other clusters in the center.

### 3.6. The most relevant scientific research institutions

**Figure 5.**
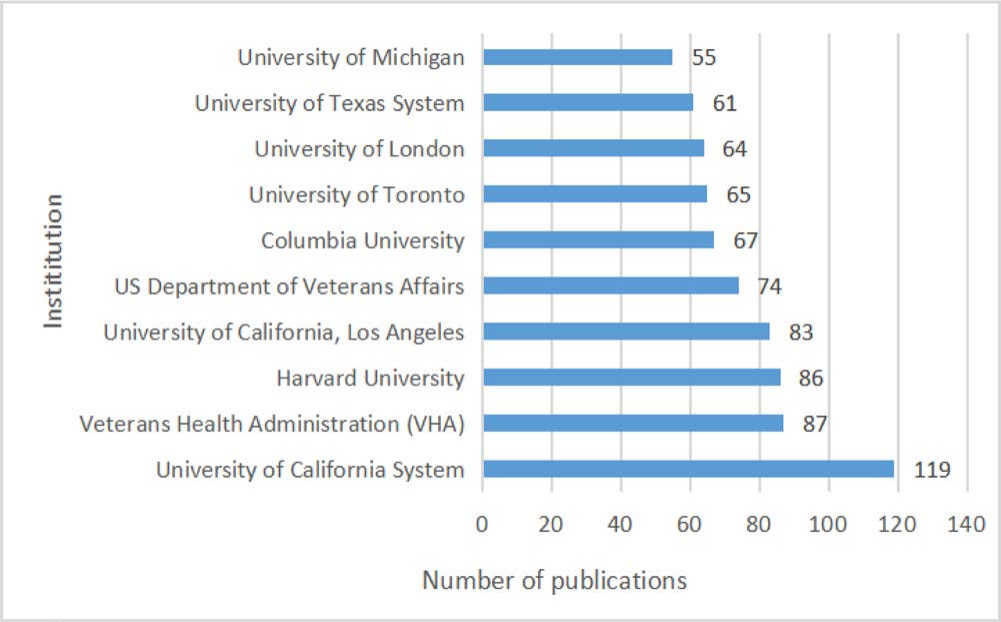
Number of articles by institutions.

The distribution of research output related to the topic of interest by research institutions is shown in Figure 5. The University of California System has published the largest number of papers in the last 20 years, with a total of 119 papers, while the output of other institutions varies widely.

### 3.7. Institutional and international collaboration networks

**Figure 6.**
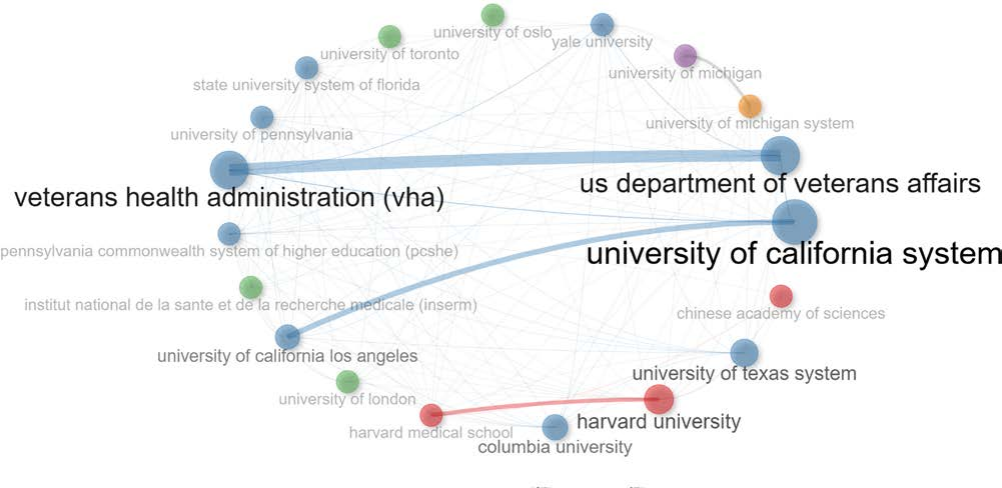
Co-authorship analysis (institution level).

**Figure 7.**
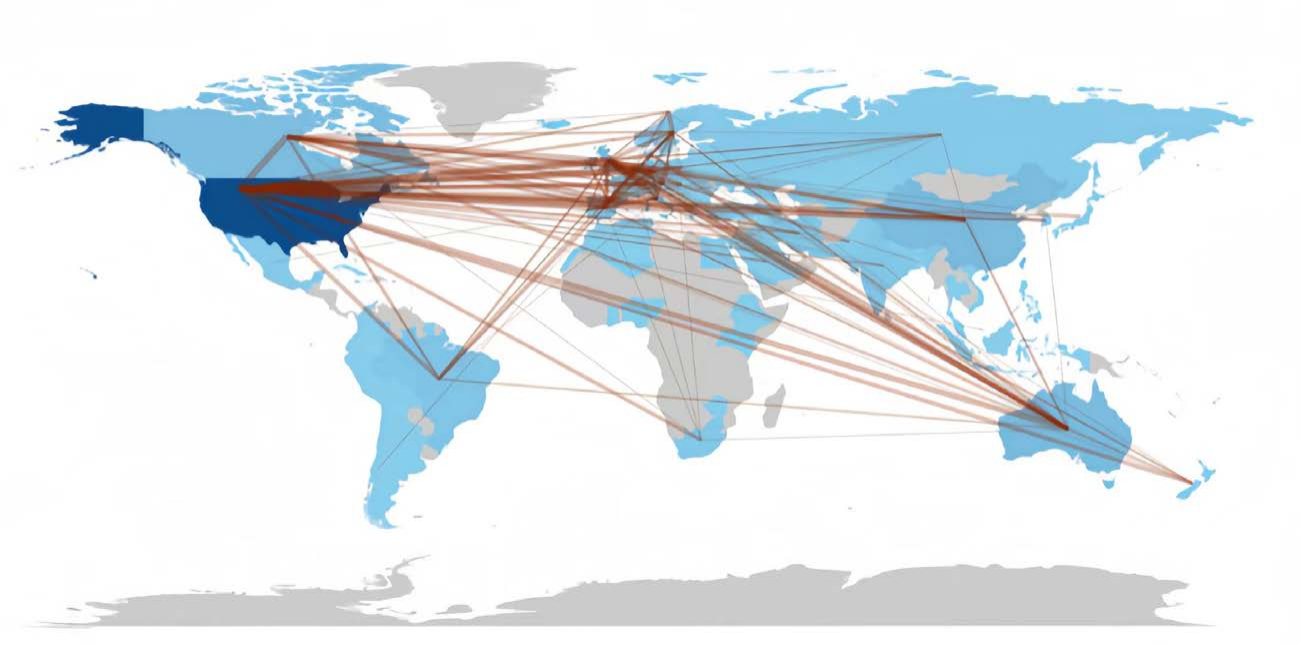
Co-authorship analysis (country level).

The collaborations between institutions are illustrated in Figure 6. Each circle represents an institution and the size of the circle represents an institution corresponds to the number of coauthored documents. The thickness of the lines between the circles represents the strength of the collaboration between the institutions. Based on the analysis, the University of California was found to have the most comprehensive network of collaborations with other institutions, thus indicating that it plays a critical role in studying the role and impact of exercise in substance abuse. Figure 7, on the other hand, represents the collaboration network of individual countries. The darker the color of the country or territory, the higher the number of published studies. Additionally, the number of lines between the countries or territories on the map represents the density of international collaborations. Based on the analysis, it was found that the United States has research collaborations with most countries. The 3 countries that have collaborated the most with the United States are the United Kingdom with 37 articles, Canada with 34 articles, and China with 25 articles.

### 3.8. Keyword co-occurrence analysis and hot trends

**Figure 8.**
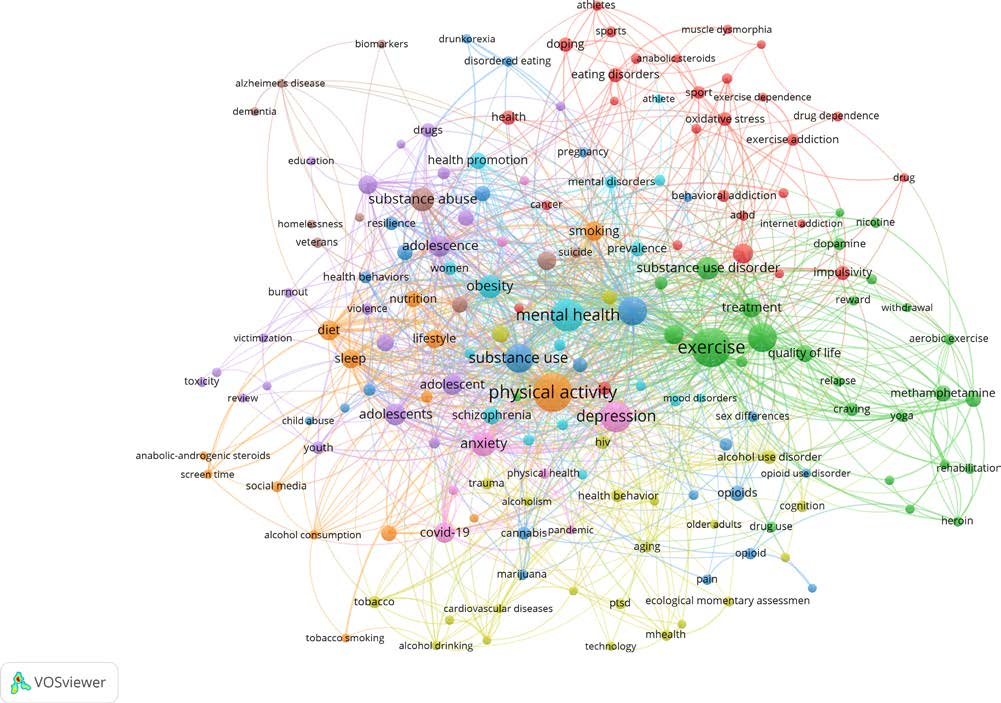
Keyword co-occurrence.

The results from the keyword co-occurrence analysis are visualized in Figure 8. There are 222 nodes in the graph, each representing a keyword. The size of the node corresponds to the frequency of the keyword used. It was found that the keywords, “exercise,” “substance abuse,” “SUDs,” “mental health,” and “depression” dominated the research in this field.

**Figure 9.**
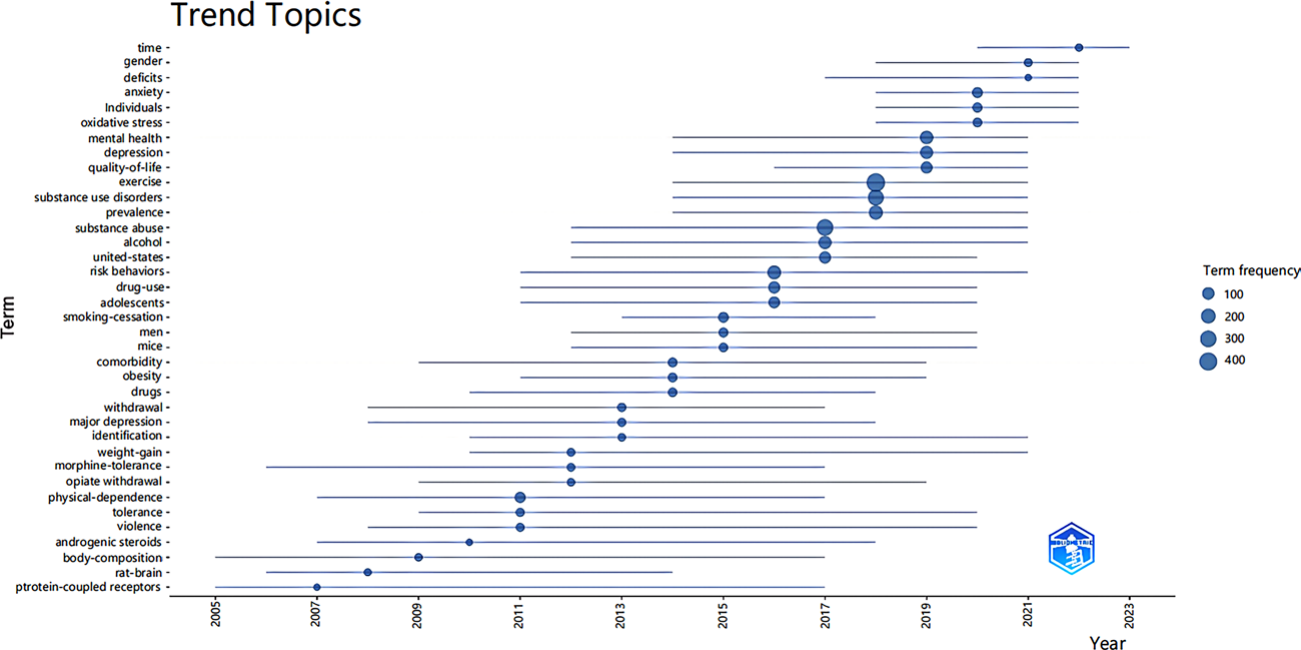
Trending topics.

Figure 9 provides insights into the evolving trends in the field of substance over the past 20 years. The graph depicts the subject’s position on the timeline, with line length indicating the duration of relevance for specific “hot” words. Larger nodes represent frequently appearing terms.

Long-studied topics include physical dependence, risky behaviors, comorbidities, and SUDs, which have remained on the research agenda for many years. However, recent trends reveal emerging issues such as exploring the intersections of SUDs with mental health issues, and gender-specific factors and investigating temporal patterns and changes in SUD-related phenomena.

### 3.9. Topic clustering and topic evolution

**Figure 10.**
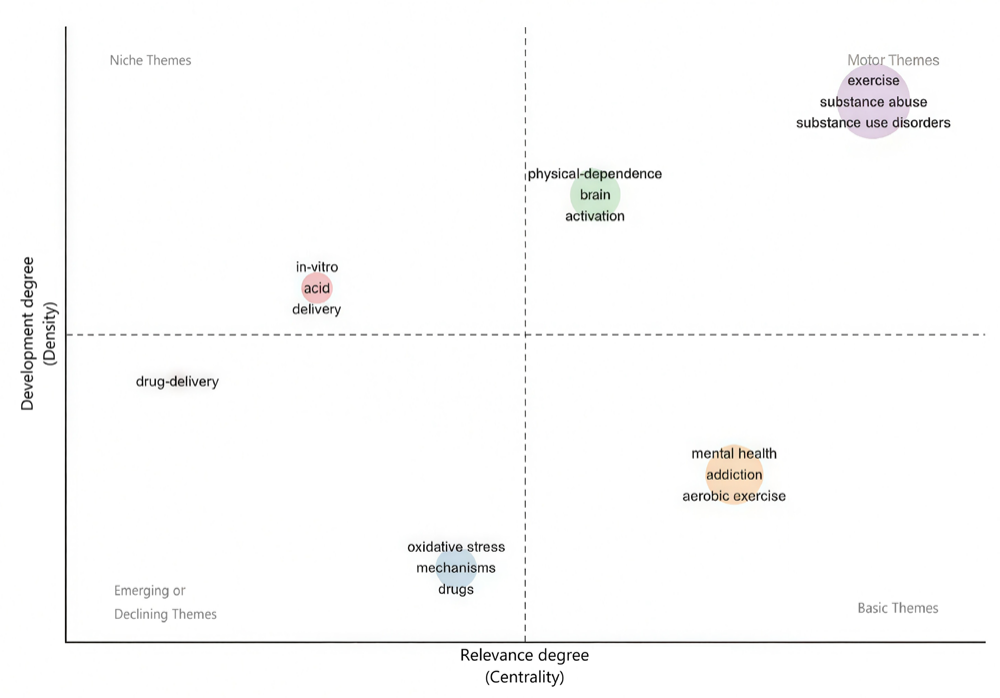
Thematic map.

**Figure 11.**
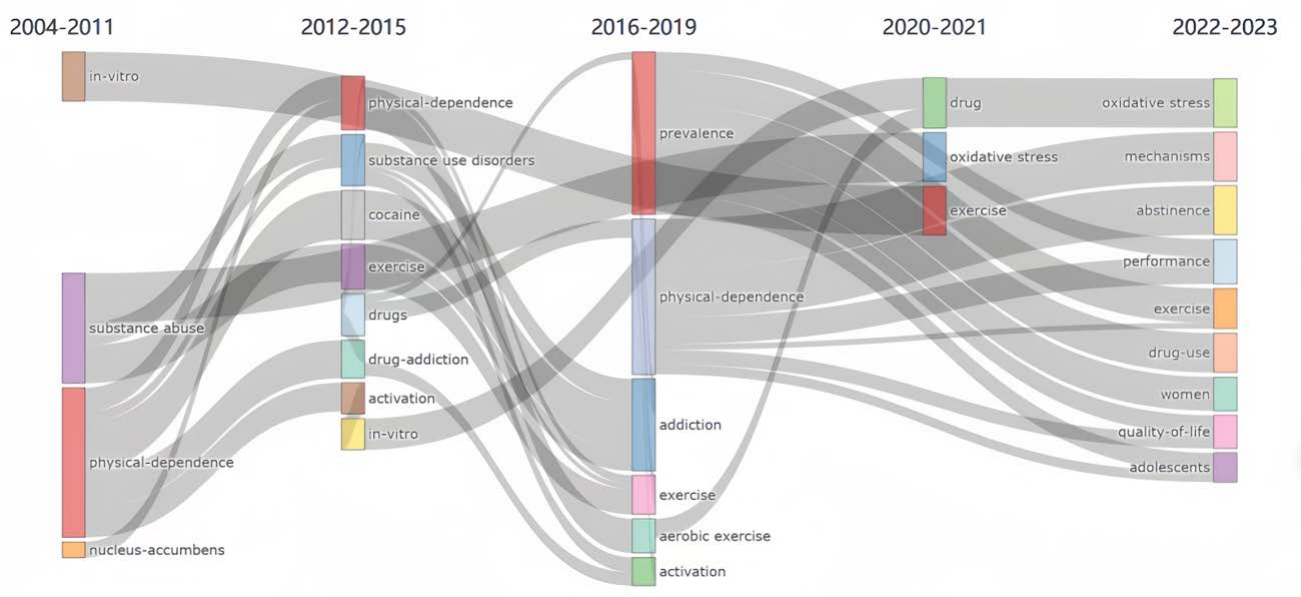
Thematic evolution.

Using cluster analysis (Fig. 10), it was found that relevant research in this field from the past 20 years can be divided into 6 thematic clusters, which include drug delivery, *in vitro*, physical dependence, exercise, and mental health. Topic evolution in publications (Fig. 11), was analyzed to understand past research trends to predict future research trends. Topics were observed to evolve from *in vitro*, substance abuse, physical dependence, and nucleus-incumbency in 2004 to oxidative stress, mechanisms, abstinence, performance, exercise, drug use, women, quality of life, and adolescents.

## 4. Discussion

Exercise as a form of intervention for substance abuse is emerging research of interest within the field. It is prescribed for detoxification,^[28]^ to alleviate withdrawal symptoms,^[6]^ and to inhibit relapse impulse and relapse behavior.^[29]^ In the past 2 decades, research on exercise for substance abuse has significantly increased in-depth and breadth, aligning with current trends and providing additional insights and methods for the treatment of substance abuse. This study analyzes the research hotspots, frontiers, and developmental trends of exercise in the treatment of substance abuse in recent years.

According to the information from the number of publications, it was found that the annual output of research publications on exercise in the context of substance abuse has been high over the past 20 years. However, the research trend showed some decline after 2021. It is possible that the global outbreak of COVID-19 has led to a decrease in published literature due to the fact that some human experiments could not be carried out. The rapid and dramatic outbreak of the new crown outbreak forced the suspension or cancelation of many ongoing basic science and clinical research projects, which led to a significant slowdown in research progress. This was especially true in the area of research on experiments requiring population participation.^[30]^

Furthermore, the overall trend appears to be increasing, indicating that this field is gaining momentum globally. Reviewing authors who have contributed to this field, it was observed that Greer TL and Trivedi MH from University of Texas Southwestern Medical Center were the most prolific authors and frequently worked closely together. Several studies have looked into the association between exercise intervention and stimulant use disorders.^[31–33]^

Several journals came out as major scholarly outputs in this field, including Drug and Alcohol Dependence, Frontiers in Psychiatry, and the International Journal of Environmental Research and Public Health. However, the impact factor of these journals is relatively low, which questions the recognition that these publications are getting. It is also important to note that no major theoretical breakthrough has been recorded, speaking to the quality of these publications. Thus, the quality of research output in this field must be improved to match the high publication quantity. When the research output was compared between countries and institutions, the United States stands out as 1 of the major contributors to influential publications. Figure 4 highlights the prevalence of U.S.-based research institutions in the field of SUD, indicating their active involvement. Notably, the University of California System has made a large number of article contributions, reflecting its high-level of research enthusiasm and focus in this area. Over the past 20 years, the United States has played a leading role in SUD research productivity, surpassing Asian and European countries. Academic collaboration is robust globally, with the United States at the center. Not only does it publish the highest number of articles, but it also maintains collaborative relations with most countries, solidifying its core position in this field. Several factors contribute to the United States’ recognition as a research leader in this field. Firstly, the incidence of SUDs has steadily risen, making it a major public health concern. In particular, regions like Appalachia, New England, Florida, and parts of Kentucky face dire situations, creating an urgent need for effective intervention. As such, the U.S. federal, state, and local governments have implemented intervention strategies to reduce SUD-related mortality, including prescription drug monitoring programs (PDMPs)^[34]^ and the Project Towards No Drug Abuse (TND).^[35]^ Adequate research funding has supported high-level scientific investigations, resulting in a strong academic reputation and high H-index values.^[36]^

Keywords are concise highlights of the research focus in literature and tracking keyword evolution helps understand the current research hot spots and helps predict the research interest trajectory. As far as mechanisms are concerned, oxidative stress has become a research focus in recent years. For example, Scheffer et al^[37]^ indicated that engaging in repetitive moderate-intensity physical activity can bolster the immune system, augment antioxidant capacity, mitigate damage from oxidative stress, and improve energy production efficiency. This drastically reduces the occurrence of inflammatory diseases. Exercise training restores the balance between excitatory and inhibitory neurotransmitters, as well as between pro- and anti-inflammatory cytokines, and reduces oxidative stress in the paraventricular nucleus.^[38]^ Based on the most frequently cited literature, it was found that in the past 20 years, the research content in this field has primarily focused on different exercise methods as an intervention for drug addicts. Current research has largely confirmed that exercise can modulate dopamine (DA) and endogenous opioid peptide (EOP) systemic disorders after substance abuse, increase the levels of oxytocin and brain-derived neurotrophic factor, and enhance the function of the neuroimmune system. Consequently, it improves the withdrawal symptoms of opioid addiction.^[39]^ The exact biological pathways by which exercise interventions influence substance abuse remain unclear, although studies have proposed mechanisms for neurotransmitter actions in these relevant brain regions. Future studies could explore these mechanisms using multi-omics approaches such as neuroimaging techniques, biomarkers and genomics.

Although exercise is recognized for its salutary effects on both physiological and psychological health of substance abusers,^[8]^ in-depth studies are still needed on the effects and mechanisms of different types of exercise, exercise duration and intensity on different genders, ages and types of substance abusers. Future research could delve more deeply into the specific biological mechanisms involved in different exercise conditions, and in particular how exercise interventions can be optimized to maximize their benefits in substance abusers. Attention also needs to be paid to the applicability of exercise interventions in different age, gender and cultural contexts in order to develop more comprehensive and individualized treatment programs. In addition, exploring the conditions of adjuvant therapy and elucidating its mechanism is also a hot topic for future research. According to the available study,^[40]^ exercise combined with medication can have a cost-saving effect and increase treatment outcomes. The mechanism of interaction between exercise as an adjunctive treatment for substance abuse and its medications is still not known. Future research could be conducted in this research direction to fill this gap.

Mental health issues related to over-dependence on 1 or more drugs, clinically known as SUD, is a common occurrence.^[41]^ The onset of mental health issues increases the long-term risk of developing serious health problems, such as reduced physical function, complex mental disorders,^[42]^ and affective disorders.^[43]^ Additionally, it is common for multiple disorders such as depressive disorder and major depressive disorder (MDD) to occur simultaneously with SUD.^[44]^ Compared to patients with only a diagnosis of MDD, patients with SUDs, in addition to MDD, have significantly more severe depressive symptoms, worse prognosis, severe functional impairment, worse recovery rates, higher morbidity and mortality, and increased frequency of hospitalizations.^[45,46]^ Treating psychological problems in SUD patients remains a challenge. Future research needs to investigate the efficacy of personalized exercise therapy in treating the mental health issues related to SUDs.

This study analyzed the current research landscape related to exercise in substance abuse recovery to be able to predict the future research trajectory in the field of motor intervention SUDs. However, there are certain drawbacks to this study. Firstly, drawing from other bibliometric studies, it is believed that the Web of Science core dataset is a reputable and reliable database that covers the vast majority of publications and citation indexes.^[47]^ Hence, data for this study was extracted only from the Web of Science and did not include other databases such as Scopus and PubMed. However, there is a possibility that biases have been introduced into this 1-sided analysis. For example, most of the included publications are in English, which might have introduced linguistic bias. Another limitation is that in this study, literature and reviews obtained from databases were identified as included in bibliometric analyses to clarify the current state of research on exercise in the substance abuse literature, while books and book chapters were not included. Therefore, the results of the study should be interpreted with caution.

## 5. Conclusion

The comprehensive bibliometric analysis of this study maps the trajectory of academic development in this research area, identifies key research themes and gives appropriate recommendations for the direction of future research. Firstly, the physiological mechanisms linking exercise to SUDs recovery remain an area ripe for exploration. While evidence suggests modulation of neurotransmitter systems and amelioration of withdrawal symptoms, the specific biological mechanisms of exercise intervention in SUDs requires further elucidation. Multi-omics approaches should be prioritized to uncover the intricate pathways through which exercise confers its therapeutic effects. Secondly, there is a clear need for more in-depth studies on the effects of different types of exercise, duration, and intensities on various substance abusers groups, as well as the optimization of exercise interventions for maximum benefit. Thirdly, addressing the psychological comorbidities of substance abuse is a pressing concern, with depression and anxiety often complicating the recovery journey. The role of exercise in mitigating these comorbidities presents a promising avenue for research, with potential implications for developing adjunctive therapies to traditional treatment modalities. In conclusion, the role of exercise in substance abuse treatment stands at the cusp of a new era, with the potential to revolutionize recovery strategies. As the field advances, it must embrace a global perspective, integrate diverse methodological approaches, and prioritize research that elucidates the intricate interplay between exercise, physiology, and psychological health.

## Acknowledgments

The authors would like to express their gratitude to the participants and staff involved in data collection and management in the database.

## Author contributions

**Conceptualization:** Jiawei Chen.

**Data curation:** Liu Sun, Tatjana A. Shilko, Jiawen Li, Qingyuan Wang.

**Investigation:** Liu Sun.

**Methodology:** Liu Sun, Tatjana A. Shilko, Xiaolou Tian.

**Supervision:** Linan Zhang.

**Validation:** Ying Tian.

**Visualization:** Ying Tian.

**Writing – original draft:** Jiawei Chen.

**Writing – review & editing:** Xing Wang, Linan Zhang.
